# Induction of Macrophage Chemotaxis by Aortic Extracts from Patients with Marfan Syndrome Is Related to Elastin Binding Protein

**DOI:** 10.1371/journal.pone.0020138

**Published:** 2011-05-27

**Authors:** Gao Guo, Petra Gehle, Sandra Doelken, José Luis Martin-Ventura, Yskert von Kodolitsch, Roland Hetzer, Peter N. Robinson

**Affiliations:** 1 Institute for Medical and Human Genetics, Charité-Universitätsmedizin Berlin, Berlin, Germany; 2 Deutsches Herzzentrum Berlin (DHZB, German Heart Institute Berlin), Berlin, Germany; 3 Marfan-Zentrum des DHZB und der Charité, Berlin, Germany; 4 Max-Planck-Institute for Molecular Genetics, Berlin, Germany; 5 Berlin Center for Regenerative Therapies (BCRT), Charité-Universitätsmedizin Berlin, Berlin, Germany; 6 Vascular Research Laboratory, Instituto de Investigacion Sanitaria, Fundación Jimenez Diaz, Autonoma University, Madrid, Spain; 7 Centre of Cardiology and Cardiovascular Surgery, Department of Cardiology/Angiology, University Hospital Hamburg–Eppendorf, Hamburg, Germany; University of Illinois at Chicago, United States of America

## Abstract

Marfan syndrome is an autosomal dominantly inherited disorder of connective tissue with prominent skeletal, ocular, and cardiovascular manifestations. Aortic aneurysm and dissection are the major determinants of premature death in untreated patients. In previous work, we showed that extracts of aortic tissues from the mgR mouse model of Marfan syndrome showed increased chemotactic stimulatory activity related to the elastin-binding protein. Aortic samples were collected from 6 patients with Marfan syndrome and 8 with isolated aneurysms of the ascending aorta. Control samples were obtained from 11 organ donors without known vascular or connective tissue diseases. Soluble proteins extracted from the aortic samples of the two patient groups were compared against buffer controls and against the aortic samples from controls with respect to the ability to induce macrophage chemotaxis as measured using a modified Boyden chamber, as well as the reactivity to a monoclonal antibody BA4 against bioactive elastin peptides using ELISA. Samples from Marfan patients displayed a statistically significant increase in chemotactic inductive activity compared to control samples. Additionally, reactivity to BA4 was significantly increased. Similar statistically significant increases were identified for the samples from patients with idiopathic thoracic aortic aneurysm. There was a significant correlation between the chemotactic index and BA4 reactivity, and the increases in chemotactic activity of extracts from Marfan patients could be inhibited by pretreatment with lactose, VGVAPG peptides, or BA4, which indicates the involvement of EBP in mediating the effects. Our results demonstrate that aortic extracts of patients with Marfan syndrome can elicit macrophage chemotaxis, similar to our previous study on aortic extracts of the mgR mouse model of Marfan syndrome (Guo et al., *Circulation* 2006; 114:1855-62).

## Introduction

Marfan syndrome (MFS) is an autosomal dominant inherited disorder of connective tissue that is caused by mutations in the gene for fibrillin-1 (*FBN1*), with prominent clinical manifestations in the cardiovascular, skeletal and ocular systems. The high mortality of untreated patients results almost exclusively from cardiovascular complications such as acute aortic dissection or rupture [1], [2]. The pathogenesis of MFS is complex, and numerous classes of abnormalities have been shown to result from mutation in *FBN1* [3]. Fibrillin-1 contributes to the sequestration of TGF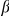 in the extracellular matrix (ECM) and thereby to the control of its bioavailability [4], [5]. Mutation in fibrillin-1 leads to increased TGF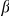 signaling activity [6]–[9]. Additionally, a number of other aspects of the molecular pathomechanism of MFS have been characterized in recent years, including evidence that haploinsufficiency for fibrillin-1 contributes to failed microfibrillar assembly and the development of disease [10], [11], endothelial dysfunction and compromised eNOS/Akt signaling [12]–[14], and alterations in the biosynthesis of fibrillin-1 rich microfibrils [15], [16].

Another line of research has examined the roles of proteases and protein fragments in MFS. A number of groups have shown that *FBN1* gene mutations can increase the susceptibility of fibrillin to proteolysis [17]–[23]. Missense mutations affecting either highly conserved cysteine residues or residues of the calcium-binding consensus sequence are common in MFS [24]. Presumably, such mutations affect the structure and conformation of the cbEGF module or cause alterations in interdomain flexibility [25], [26] and thereby expose the modules to proteases. There is histological evidence of fragmentation [27], [28] in addition to evidence of alterations in matrix metalloproteinase (MMP) and tissue inhibitor of MMP (TIMP) activity [29]–[31] in the aortic tissues of Marfan patients. Another indication of the potential importance of altered protease activity for the pathogenesis of MFS is the observation that treatment of mice with mutations in the *Fbn1* gene with doxycycline, a non-specific MMP inhibitor, significantly delays aneurysm rupture in MFS-like mice by inhibiting expression of tissue MMP-2 and MMP-9 and thus, degradation of the elastic matrix [32], [33].

The fourth LTBP domain of fibrillin-1 contains an Arg-Gly-Asp (RDG) integrin-binding motif that mediates binding to several integrins and thereby plays a role in adhesion and migration of cells [34]–[39]. Fibrillin-1 additionally contains three Gly-x-x-Pro-Gly (GxxPG) motifs similar to a repeated peptide in elastin, Val-Gly-Val-Ala-Pro-Gly (VGVAPG), is known for its chemotactic activity to fibroblasts and monocytes [40] This effect is mediated by binding to the 67-kDa elastin binding protein (EBP) present on the surface of mononuclear phagocytes. Elastin-derived peptides (EDPs) released from human AAA tissue can attract mononuclear phagocytes through ligand-receptor reactions with the EBP [41].

In previous work, we showed that fibrillin-1 fragments containing the RGD or one of the GxxPG motifs can upregulate MMP activity in cell culture [42], [43]. This led us to investigate whether ascending aortic samples from the fibrillin-1 underexpressing mgR mouse model for MFS can act as chemotactic stimuli for macrophages. Both the aortic extracts from the mgR/mgR mice as well as a GxxPG-containing fibrillin-1 fragment significantly increased macrophage chemotaxis compared with extracts from wild-type mice or buffer controls. The chemotactic response was significantly diminished by pretreatment of macrophages with lactose or with the elastin-derived peptide VGVAPG and by pretreatment of samples with a monoclonal antibody directed against an EBP recognition sequence. Additionally, investigation of macrophages in aortic specimens of Marfan patients demonstrated macrophage infiltration in the tunica media [44].

The aim of the present study was to investigate whether aortic extracts from human patients with MFS demonstrate chemotactic stimulatory activity as previously demonstrated for the mgR mouse model of MFS. A total of 6 patients with MFS and 8 with isolated thoracic aortic aneurysm (TAA) were investigated.

## Materials and Methods

### Patient Selection and Preparation of human aortic extracts

Fresh full-thickness aorta specimens were obtained from patients at the time of aortic replacement or cardiac transplantation at the German Heart Institute Berlin between the years 2008 and 2010. Inclusion criteria were MFS (n = 6) and isolated TAA (n = 8). Additionally, aortic samples from eleven heart donors with no known cardiovascular disease were obtained at the time of heart transplantation in the operating theater during the time of implantation of the donor heart into the recipient. The cooled ischemic time was never longer than 3 hours, and therefore, the aortic specimens had been on ice for maximally 3 hours at the time the specimens were taken. The diagnosis of MFS was based on Ghent criteria [45] (The study was completed prior to the publication of the recently revised version of the Ghent nosology [46]). In 5 of the Marfan patients, the diagnosis was additionally confirmed by genetic mutation analysis.

Tissue samples (about 0.5×2 cm) were taken during surgery after the initialization of cardiopulmonary bypass during cross-clamp time and immediately following the aortotomy. Samples were excised from the ascending aorta just above the sino-tubular junction. Tissue samples of donor hearts were cut off during ischemic time just before the implantation of the donor heart at transplantation. The specimens were immediately placed into phosphate-buffered saline (PBS) solution. Then they were stored at 4°C until they were sent to the laboratory on the same day.

Aortic tissue extracts were prepared using a modification of a previously described protocol [41]. Briefly, the adventitia was dissected from aorta, tissue specimens comprising the medial and intimal layers were washed 2 times in PBS and weighed. Specimens were then incubated in extraction buffer (PBS with 2 mol/L NaCl) overnight at 4°C with gentle shaking (20 ml per 1 g tissue). At the end of incubation, aortic tissue was removed, and the incubation solution was centrifuged at 10 000 rpm for 30 minutes to remove particulate debris. Aortic extract solution was stored at 4°C for experiments on the following day (or at 20°C for future repeats). Total protein concentration was measured by a BCA assay kit (Pierce). Aortic specimens were divided according to their diagnosis. Aortic extracts used for chemotaxis assay were diluted in PBS to make a final concentration of 20 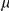
g/ml of total protein.

The study was approved by the ethics commission of the Charité Campus Virchow-Klinikum (EA2/096/07).

### Chemotaxis Assay

The murine macrophage cell line RAW 264.7 (ATCC TIB-71) was cultured in medium containing RPMI 1640 (Biochrom AG, Berlin, Germany), 10% FBS, and penicillin/streptomycin mix (final concentration: penicillin, 100 IU/ml; streptomycin, 100 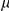
g/ml). RAW 264.7 cells have properties similar to those of mouse resident macrophages and exhibit responsiveness to chemotactic stimuli [47].

For harvesting the cells, monolayers of RAW 264.7 cells were washed 2 times with PBS and then with a nonenzymatic cell dissociation solution (Sigma-Aldrich). After 25 minutes of incubation at room temperature, cells were suspended in PBS, counted, centrifuged, and finally suspended in the chemotaxis buffer consisting of RPMI 1640 supplemented with 1% bovine serum albumin (BSA, Sigma-Aldrich) at 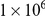 cells/ml.

The chemotaxis assay was performed in a 48-well chemotaxis chamber (NeuroProbe). The bottom wells were filled with 25 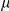
l of attractant solution. An 8-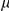
m-pore-diameter polyvinylpyrrolidone-free polycarbonate filter (NeuroProbe, Gaithersburg, MD, USA) was placed on the bottom plate. A silicon gasket and the top plate with 48 holes were then mounted, forming the top wells. The cells were added in a volume of 50 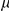
l. In parallel experiments, extraction buffer was loaded into the bottom wells as a negative control. After 2 hours of incubation at 37°C and 5% CO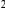, the filter sheet was removed, and nonmigrated cells were wiped off the top side. The filter was then fixed in 70% methanol for 30 seconds and stained in Wright-Giemsa (Sigma-Aldrich).

### Competitive ELISA

GxxPG containing fragments concentration was measured by using a competitive ELISA methods as described previously by Wei et al.[48], with some modifications. Maxisorb 96-well microtiter plates (Nunc) were coated with 50 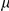
l of elastin peptide (0.5 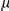
g/ml, CB573, Elastin Products Company) in PBS, pH 7.4 and incubated overnight at 4°C. The wells were blocked with 100 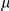
l of 0.5% BSA in PBS containing 0.05% Tween 20 (PBS-T). Spike-and-recovery experiment detects a discrepancy between standard diluent (PBS) and 2 fold diluted extraction buffer (Data not shown). Therefore, in oder to generate a standard curve, a variable concentration of elastin peptides from 10 ng/ml-10 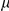
g/ml diluted in 1∶2 extraction buffer is mixed with a 1∶1000 dilution of the mouse monoclonal antibody derived from immunization against bovine 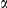-elastin (mAb BA4, Sigma-Aldrich) and incubated in a 0.5% BSA precoated plate. The standard curves were fitted using a four-parameter logistic model. Simultaneously, 25 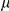
l of each sample was diluted 2 times with PBS and were mixed with 50 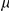
l of 1∶1000 diluted BA4 antibody. After overnight incubation with mild shaking at 4°C, 100 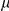
l mixtures were then added to each well in the elastin peptide coated plate and incubated for 30 min at 37°C, the plates were washed three times with PBS-T, followed by the addition 50 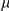
l of secondary antibody (1∶2000 anti-mouse IgG peroxidase conjugate). After 1 hour incubation at 37°C, the plates were washed three times, 50 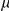
l of tetramethylbenzidine substrate solution (Thermo Fisher Scientific, San Jose, CA) was added, and after 10 minutes incubation at room temperature the reaction was quenched by adding 50 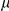
l 1 M H
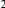
SO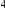 to each well. The absorbance was measured at 450 nm using a micro-plate reader. The accuracy and precision of the quantitative range of the ELISA was determined by replicate analyses. The concentration of GxxPG containing fragments was normalized against the total protein concentration of the aortic extracts.

### Experimental Design

Aortic extracts from patients with MFS and isolated TAA were compared against control aortic extracts derived from heart donors with no known cardiovascular disease. Our previous work on the chemotactic stimulatory potential of aortic extracts from the mgR Marfan mouse model had suggested that the chemotactic activity of aortic extracts is at least partially mediated by EBP [44]. Therefore, we performed several experiments to investigate whether the chemotactic activity of human aortic samples is also mediated by the EBP.

BA4 can block the chemotactic activity of VGVAPG, a repeated amino acid sequence motif in elastin that is know to bind to the EBP [41]. Preincubation of monocytes with VGVAPG peptides can also block the chemotactic response [40]. The EBP is known to dissociate in the presence of high levels of lactose, but not glucose [49]. Therefore, to investigate a potential role with EBP in the chemotaxis experiments described in this manuscript, cells were exposed to 1 mmol/L lactose or glucose, or 0.1 mmol/L VGVAPG for 1 hour incubation at 37°C before the chemotaxis assays were started. As a further control, samples were preincubated for 30 minutes at room temperature with BA4 or with murine IgG (Sigma-Aldrich), both at a dilution of 1∶1000. All assays were done in triplicate during the same experiment. In each well, 4 random fields were chosen with the microscope set at x200 magnification. The fields were observed and recorded photographically with the Leica DC viewer program. Fields were scored by 2 observers blinded to the chemotactic stimulus (or control) using an in-house Java program. PBS was loaded into the bottom wells as a negative control. The results were expressed as the chemotactic index (CI) that is the number of cells having migrated in the presence of the chemotactic agent divided by the number of cells having migrated in the presence of extraction buffer alone.

### Statistical analysis

Data shown represent the mean
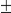
SD (standard deviation). The value for the mean BA4 reactivity and chemotactic index (CI) of each group was compared using the Student’s *t* test. Additionally, Fisher’s exact test was performed to compare the gender distribution in the two patients groups, and the Mann-Whitney test was performed to compare age, CI and BA4 reactivity between two patient groups. Statistical significance was accepted at *P* 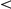
0.05.

## Results

The mgR mouse underexpresses fibrillin-1 and recapitulates many of the clinical features of human MFS [50]. In previous work, we showed that aortic extracts of the mgR mouse induce macrophage chemotaxis. The chemotactic inductive effect could be inhibited by pretreating the macrophages with lactose, which causes shedding of the elastin-binding protein, or by pretreatment of the aortic extracts with the monoclonal antibody BA4, which reacts against bioreactive GxxPG motifs in elastin [51] and, as we showed, against a GxxPG motif in fibrillin-1 [44]. In this work, we asked whether aortic extracts from human Marfan patients would similarly induce macrophage chemotaxis and whether evidence could be obtained that this effect is at least partially mediated by EBP.

### Clinical data

Six specimens were obtained from individuals with MFS and eight specimens were obtained from individuals with isolated TAA. Four of the Marfan patients were receiving prophylactic treatment with beta blockers at the time of the operation. Additionally, samples from 11 heart donors with no known cardiovascular disease were used as controls. The average age of the patients with MFS was 38.5
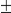
16.1 years, and five of the six patients were male. The average age of the patients with isolated TAA was 58.6
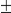
12.1 years, and all eight patients were male. Details of patient characteristics are shown in Table 1. There is a significant difference regarding age between MFS and TAA group (Table 2).

**Table 1:** Characteristics of the patients included in this study.

Patient	Diagnosis	Age	Sex	Description
1	MFS	23	m	Dilatation and dissection of the ascending aorta and abdominal aorta, maximum diameter of ascending aorta 4 cm. Atenolol.
2	MFS	45	m	Aneurysm of ascending and descending aorta, status post previous surgical repair of ascending aorta with leakage of anastomosis.
3	MFS	21	f	Sinus valsalvae aortic aneurysm, status post previous surgical repair of ascending aorta. Metoprolol.
4	MFS	65	m	Infrarenal aortic aneurysm, maximum diameter 6.1 cm. Status post previous surgical repair of ascending aorta. Nebivolol.
5	MFS	37	m	Thoraco-abdominal aneurysm, status post previous surgical repair of ascending aorta. Nebivolol.
6	MFS	40	m	Dilatation of ascending aorta
7	TAA	62	m	Aneurysm of ascending aorta, III  aortic insufficiency. Metastatic colon cancer
8	TAA	40	m	Subacute dissection of the ascending aorta, status post previous surgical repair of ascending aorta, Erdheim-Gsell media degeneration, maximal diameter of sinus valsavae 4.1 cm. *FBN1* mutation excluded.
9	TAA	65	m	Aneurysm of ascending aorta, maximal diameter 6.3 cm, three-vessel coronary artery disease, arterial hypertension, hyperlipoproteinemia, sleep apnea syndrome.
10	TAA	71	m	Acute type A dissection, maximal diameter 4.2 cm. Arterial hypertension, hyperlipoproteinemia
11	TAA	73	m	Aneurysms of ascending and abdominal aorta. Maximal diameter of ascending aorta 5.3 cm. Arterial hypertension, hyperlipoproteinemia, osteoporosis
12	TAA	60	m	Dilatation of the ascending aorta, status post surgical replacement of the aortic valve. III  atrioventricular block
13	TAA	43	m	Type A dissection, septic multiorgan failure related to fulminant pneumonia, status post surgical supracoronary aorta replacement.
14	TAA	55	m	Aneurysm of the ascending aorta. Combined vitium of the aortic valve. Arterial hypertension.

MFS: Marfan syndrome; TAA: isolated thoracic aortic aneurysm; m: male, f: female; The column *Description* provides details of the indications for aortic surgery and information about other relevant medical conditions, and treatment with beta blockers.

**Table 2:** Comparison between the two patient groups.

	MFS	TAA	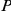 -value
Male	5	8	.429
Age(years)	39 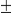 16	59 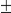 12	.020
CI	2.58 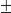 0.42	2.45 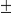 1.42	.804
BA4 reactivity ( 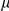 g/mg)	7.495 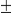 1.93	8.409 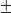 6.884	.670

The third column shows the 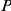-value calculated with the Fisher’s exact test for categorical data and by the Mann-Whitney test for quantitative data.

### Analytical performance of ELISA

BA4 reactivity was determined from calibration curve derived from standards of known elastin peptide concentration (from 10 ng/ml to 10 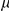
g/ml). The quantitative range of the assay was 75 ng/ml to 2 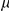
g/ml (80–20% inhibition values), with 50% inhibitory concentration at 390 ng/ml. The precision of the assay was acceptable with intra- and inter-assay coefficients of variation (CV) ranged from 2.5% to 6.3% and 2.6% to 8.7%, respectively. These results indicated that the values obtained with competitive ELISA in this study were highly reproducible and reliable.

### Higher BA4 reactivity in aortic extracts from Marfan patients

**Figure 1:**
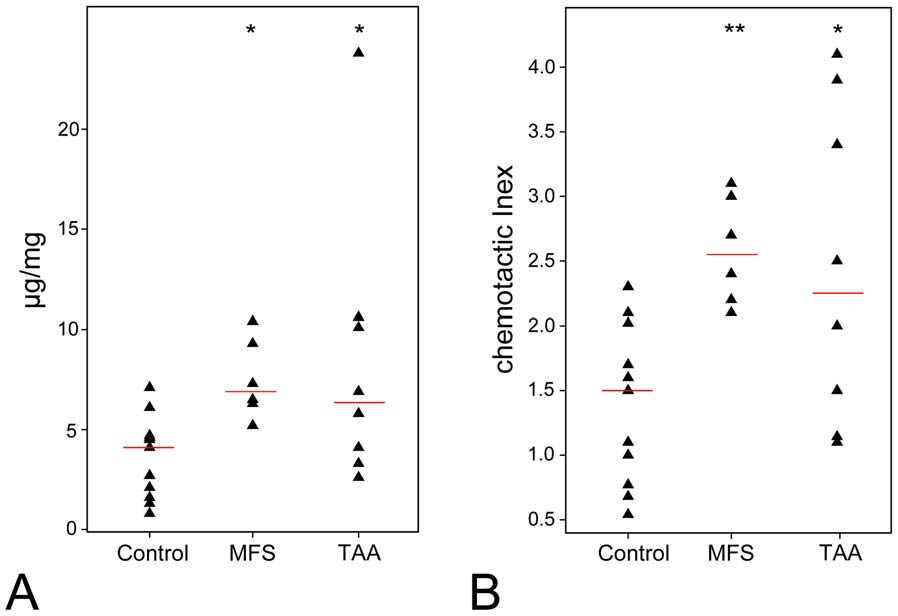
BA4 reactivity and chemotactic activity of human aortic extracts. **(A)** BA4 reactivity was measured by competitive ELISA in aortic extracts from patients with MFS (n = 6), isolated TAA (n = 8) and controls (n = 11). A statistically significant increase in BA4 reactivity as compared to control samples was observed for the samples from individuals with MFS and isolated TAA. **(B)** Chemotactic activity of the same extracts was measured by a Boyden chamber. A statistically significant increase in chemotactic activity as compared to control samples was observed for the samples from individuals with MFS and isolated TAA. Red lines indicate the median levels of BA4 reactivity or chemotactic index (CI). Data are representative of three independent experiments. * 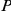 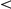
0.05, **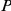 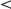
0.01.

We first asked whether the concentration of GxxPG fragments (defined operationally as reactivity against the BA4 antibody) was increased in specimens from individuals with MFS. As described in methods, we developed an ELISA assay based on the BA4 antibody to measure GxxPG containing fragments. The BA4 reactivity was significantly higher in Marfan patients (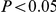) and also in patients with isolated TAA (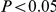) compared with control specimens (Figure 1A).

### Aortic extracts from Marfan patients demonstrate chemotactic activity

**Figure 2:**
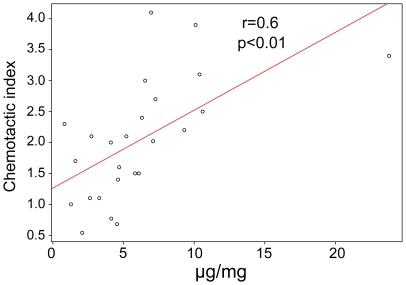
BA4 reactivity (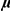
g/mg) versus chemotactic index (CI) of human aortic extracts, with calculated Pearson correlation coefficient (r) and 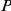 value.

We then used aortic extracts from the same samples to investigate a potential induction of macrophage chemotaxis. Extracts from Marfan patients showed significantly higher chemotactic activity (CI = 2.58
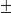
0.42, 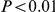) than that of 11 control samples (CI = 1.42
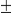
0.61) (Figure 1B). The maximal RAW 264.7 migration upon stimulation by aortic extracts from individuals with MFS was 110% of that observed upon stimulation with 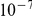 mmol/L VGVAPG. The CI of RAW 264.7 cells toward extracts from patients with isolated TAA was significantly higher (CI = 2.45
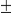
1.22, 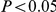) compared with control samples (Figure 1B), and maximal RAW 264.7 migration upon stimulation by extracts from TAA group was around 135% of that with 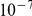 mmol/L VGVAPG. There was a statistically significant positive correlation (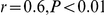) between BA4 reactivity and CI (Figure 2).

**Figure 3:**
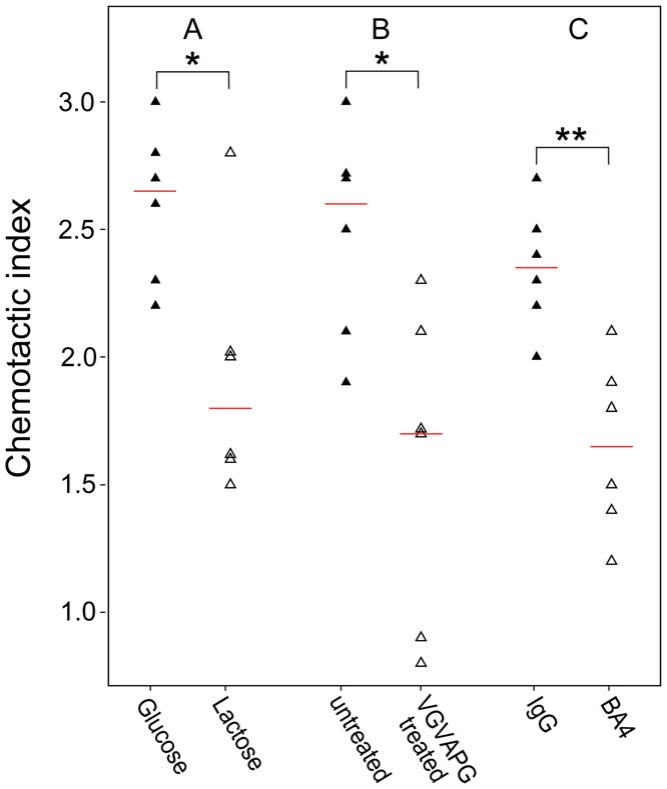
Chemotactic index (CI) of RAW 264.7 cells upon stimulation with aortic extracts from individuals with MFS (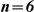). **(A)** RAW 264.7 cells were preincubated with 1 mmol/L lactose or glucose for one hour at 37°C prior to exposure to aortic extracts. There was a statistically significant inhibition of chemotaxis. **(B)** RAW 264.7 cells were preincubated with 0.1 mmol/L VGVAPG hexapeptide for 1 hour incubation at 37°C before the chemotaxis assays were started. There was a statistically significant inhibition of the chemotactic response after VGVAPG pretreatment. **(C)** Aortic extracts were preincubated with BA4 or non-specific IgG for 30 minutes at room temperature prior to chemotaxis assays. There was a statistically significant inhibition of the chemotactic response by BA4 pretreatment. Data are representative of three independent experiments. * 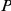 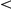
0.05, **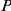 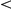
0.01.

To investigate whether chemotactic inductive activity of aortic extracts from patients with MFS is related to EBP, chemotaxis analysis was performed as described in methods. As the results shown chemotactic response of aortic extracts reduced significantly after lactose and VGVAPG pretreatment, whereas no effects were observed in cells exposed to glucose (Figure 3). Additionally, preincubation of aortic extracts with BA4 resulted in a significant reduction of the CI of RAW 264.7 in both groups (Figure 3). These results indicate that the chemoattractive ability of extracts from patients with MFS is at least partially dependent on EBP.

## Discussion

The results of this study show that extracts from the ascending aorta from patients with MFS possess increased reactivity for the monoclonal antibody BA4, which reacts with an epitope that has been mapped to VGVAPG in elastin [43], [52]. The samples additionally showed a statistically significant ability to induce macrophage chemotaxis in a Boyden chamber assay (Figure 1). Because of the fact that the chemotaxis inductive effect could be inhibited by pretreatment of the cells with lactose or pretreatment of the samples with BA4, we infer that the induction of chemotaxis is at least partially due to increased concentrations of elastin and perhaps fibrillin degradation products containing an GxxPG epitope. This interpretation is further supported by the statistically significant correlation between the BA4 reactivity and CI of aortic samples (Figure 2).

Elastin is the dominant ECM protein in the tunica media of the arterial and aortic wall, which is composed of a dense population of concentrically organized vascular smooth muscle cells (vSMCs) that synthesize elastin molecules and secrete them as soluble, hydrophobic monomers termed tropoelastin. As a part of the aging process, the aortic wall undergoes a number of alterations including fibrosis and elastin fragmentation, characterized by disruption of elastin lamellae [53]. Correspondingly, the concentration of soluble elastin fragments in serum gradually increases in healthy subjects with aging [54]. Elastin fragmentation is known to be a component of the complex pathogenesis of pulmonary emphysema, which results in part from elastic tissue digestion by unrestrained elastase activity in the lung in turn leading to the release of soluble elastin fragments (EDP), which may be measured in plasma by an ELISA [55]. Elastin has multiple cell hydrophobic repeating sequences with a core sequence of GxxPG that can induce a chemotactic response [56], and EDPs have been shown to drive disease progression in a mouse model of emphysema [57].

There is some suggestive evidence that EDPs may contribute to progression of vascular diseases. Soluble EDPs released within human abdominal aortic aneurysm tissue can attract mononuclear phagocytes by interacting with the 67-kD EBP [57], this observation offers a plausible, if unproven explanation for the inflammatory response that often accompanies abdominal aneurysmal degeneration. EDPs can induce free radical and protease production and induce oxidation of low density lipoproteins by phagocytic cells [58], [59]. However, the relation of elastin and human arterial disease is complex and incompletely understood. Macrophages are not only able to be attracted by elastin fragments, but can themselves produce the elastin precursor elastin, and may contribute to the increase in tropoelastin content that is characteristic of human abdominal aortic aneurysms and atheromas [60].

In the present study, we have presented evidence that aortic extracts from patients with MFS contain increased concentrations of EDPs that react with BA4 and are able to induce macrophage chemotaxis. We had previously shown that aortic extracts of a mouse model of MFS as well as a GxxPG-carrying fibrillin-1 fragment can induce macrophage chemotaxis by a mechanism that is at least partially mediated by the 67-kD EBP [44]. The results of the current study have shown a similar phenomenon for aortic specimens from human Marfan patients. Together with the finding of a modest but statistically significant increase in the number of CD68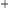 macrophages in aortic specimens of Marfan patients [44], our findings provide suggestive evidence that secondary effects of elastin fragments including the induction of macrophage chemotaxis may contribute to the complex pathogenesis of MFS. Further studies will be required to determine whether elastin fragments drive disease progression or whether they represent an epiphenomenon.

Eight patients underwent prophylactic aortic operations because of isolated (idiopathic) TAA. Several genes and genetic loci have been identified for familial TAA [61], although the etiology of most cases of isolated TAA still remains unknown. Genetic testing was not performed on the eight patients with TAA, but there were no clinical signs suggestive of known monogenic forms of TAA such as Loeys-Dietz syndrome. Given the average age of 58.6
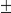
12.1 years in this group of patients, and the history of arterial hypertension in several patients, it appears likely that most if not all of these patients do not have a monogenic form of TAA. BA4 reactivity and the chemotactic index for the TAA group was statistically significantly increased, although the individual values showed a larger dispersal than did the samples from the Marfan patients.

In summary, this work has shown that aortic extracts from individuals with MFS and isolated TAA can induce macrophage chemotaxis modulated by the 67-kD EBP. This suggests that elastin fragmentation may be a common response of the damaged aorta. A limitation of our study is related to the fact that no identifying data are available for the control aortic specimens, which were obtained from deceased heart donors with no known cardiovascular disease. Therefore, confounding effects related to age or gender cannot be excluded. It remains to be determined whether secondary effects of these fragments that are modulated by the 67-kD EBP drive progression of some or all of these diseases. Since increased GxxPG epitope was observed in aortic extracts from both Marfan and TAA patients, it will be interesting to develop a more sensitive ELISA method to investigate the level of GxxPG in serum of individuals affected by MFS or other aortic aneurysm related disease.
